# Diagnostic performance of ^18^F-FDG PET-CT and SPECT for bone metastases from colorectal cancer: a retrospective study

**DOI:** 10.3389/fonc.2026.1829353

**Published:** 2026-06-12

**Authors:** Fangyi Gong, Yu Fang, Ting Ge, Yuanjin Gong, Bo Zhang, Baiwen Hu

**Affiliations:** Department of Orthopedics, The First Affiliated Hospital of Ningbo University, Ningbo, China

**Keywords:** bone metastasis, colorectal cancer, PETCT, retrospective cohort study, SPECT

## Abstract

**Objective:**

To compare the diagnostic performance of 18F-FDG PET-CT and SPECT whole-body bone imaging for detecting bone metastases in patients with colorectal cancer.

**Methods:**

A retrospective analysis was performed on 118 patients with pathologically confirmed colorectal cancer who underwent both 18F-FDG PET-CT and SPECT whole-body bone imaging within one month during follow-up for newly suspected bone metastases. The cohort included 62 males and 56 females, with a mean age of 59.1 ± 7.6 years. The final diagnosis of bone metastases was established based on comprehensive imaging follow-up, including CT, MRI, SPECT, PET-CT, and clinical progression over more than 6 months. Diagnostic performance was evaluated on a lesion-based basis.

**Results:**

A total of 533 abnormal lesions were detected in 118 patients, including 469 metastatic lesions and 64 benign lesions. PET-CT detected 423 lesions, whereas SPECT detected 398 lesions, with 288 lesions identified by both modalities. PET-CT correctly identified 428 metastatic lesions, yielding a sensitivity of 91.3%, while SPECT identified 280 metastatic lesions, with a sensitivity of 59.7%. Among the benign lesions, PET-CT correctly identified 60 lesions, resulting in a specificity of 93.8%, whereas SPECT correctly identified 30 lesions, with a specificity of 46.9%. The overall diagnostic accuracy of PET-CT and SPECT was 91.6% and 58.2%, respectively.

**Conclusion:**

18F-FDG PET-CT demonstrated significantly higher sensitivity, specificity, and diagnostic accuracy than SPECT for detecting bone metastases from colorectal cancer. PET-CT may provide superior diagnostic value for predominantly osteolytic bone metastases in colorectal cancer, while SPECT remains an important and widely available first-line screening modality in routine clinical practice.

## Introduction

Colorectal cancer is a malignant tumor that seriously threatens human health and has now become the third leading cause of cancer-related deaths worldwide (1). In recent years (2), due to the changes in risk factors for colorectal cancer and the dual effects of population growth and aging, colorectal cancer has become one of the most common and most significantly increasing malignant tumors in China (3).

With the prolongation of the survival time of patients with colorectal cancer and the advancement of imaging techniques, the incidence of bone metastasis of colorectal cancer has gradually increased (4, 5). While controlling the primary lesion, the early diagnosis and treatment of bone metastasis cannot be ignored. At present, the available evidence for reference regarding bone metastasis of colorectal cancer is very limited (6). In this background, the commonly used clinical method for diagnosing bone metastasis is SPECT whole-body bone imaging, which is recognized as a detection method with high sensitivity but low specificity for detecting bone metastasis of malignant tumors.

With the popularization of PET-CT in clinical applications, scholars have also conducted corresponding studies on its value in the diagnosis of bone metastatic cancer (7). However, the conclusions are not completely consistent, which may be related to the different natures of bone metastatic cancer in different primary tumors (8). At present, there are relatively few reports on the classification research of different primary tumors in China. This study retrospectively compared the diagnostic value of SPECT and ^18^F-FDG PET-CT whole-body imaging for bone metastasis of colorectal cancer, providing a basis for clinical application.

## Methods

### Patients

118 outpatients or inpatients in the hospital from October 2018 to February 2025 were included. There were 62 males and 56 females, aging from 48 to 75. All patients underwent routine follow-up after treatment for colorectal cancer. During follow-up, patients with newly suspected bone metastases underwent both 18F-FDG PET-CT whole-body imaging and the SPECT whole-body bone imaging. The interval between the two examinations was less than one month.

### Image analysis

All PET data were processed by image reconstruction using the OS-EM system, with a layer thickness of 3.27 cm. Two experienced physicians judged the abnormal metabolic foci of ^18^F-FDG visually and measured the SUV(maximum value)> 2.5 as a positive manifestation (SUV determination was calculated based on the patient’s weight in kilograms), or CT showed osteolytic, osteogenic or mixed changes but no increase in ^18^F-FDG metabolism, which was also regarded as an abnormal lesion. Abnormal lesions are identified as radioactive concentration foci or radioactive sparse defect foci in SPECT whole-body bone imaging. Among them, multiple abnormal radioactive concentration or sparse foci are diagnosed as bone metastasis lesions, and single lesions are classified as suspicious lesions and benign lesions based on their location, morphology and radioactive distribution (9).

### Diagnostic criteria for bone metastases

The final diagnosis of bone metastases was established based on comprehensive imaging follow-up, including CT, MRI, SPECT, PET-CT, and clinical progression over more than 6 months. No patients underwent pathological biopsy of bone lesions in this retrospective cohort (10).

### Statistical analysis

The diagnostic performance of 18F-FDG PET-CT and SPECT were evaluated mainly on a lesion-based basis, including sensitivity, specificity, and accuracy. Because multiple lesions could originate from the same patient, intra-patient correlation may exist. Considering the retrospective design and relatively limited sample size, clustered statistical analysis was not performed in the present study, which may represent a limitation of the study.

## Results

A total of 533 abnormal lesions were detected by PET-CT and SPECT in 118 patients with colorectal cancer. Among them, 423 lesions were detected by PET-CT, 398 lesions by SPECT, and 288 common lesions were found by both modalities (**Table 1**).

**Table 1:** Patient characteristics.

Variable	Value
Total patients	118
Male/Female	62/56
Mean age	59.1 ± 7.6 years
Primary tumor	Colorectal adenocarcinoma

Among the 533 lesions, 469 were finally confirmed as bone metastases. PET-CT correctly identified 428 metastatic lesions, yielding a sensitivity of 91.3%, while SPECT identified 280 metastatic lesions, with a sensitivity of 59.7% (**Table 2**).

**Table 2:** Lesion distribution.

Item	Number of lesions
Total abnormal lesions	533
Metastatic lesions	469
Benign lesions	64
Lesions detected by PET-CT	423
Lesions detected by SPECT	398
Common lesions detected by both modalities	288

Among the 64 benign lesions, PET-CT correctly identified 60 lesions, resulting in a specificity of 93.8%, whereas SPECT correctly identified 30 lesions, with a specificity of 46.9%.

The overall diagnostic accuracy of PET-CT and SPECT for bone metastases was 91.6% and 58.2%, respectively (**Table 3**).

**Table 3:** Diagnostic performance of PET-CT and SPECT.

Modality	Sensitivity (%)	Specificity (%)	Accuracy (%)
PET-CT	91.3	93.8	91.6
SPECT	59.7	46.9	58.2

## Discussion

Bones are a common site for metastasis of malignant tumors, especially colorectal cancer, breast cancer and prostate cancer, which are all highly prone to bone metastasis (11, 12). Whether malignant tumors have bone metastasis or not is of great significance for clinical staging (13), the selection of treatment plans and the evaluation of prognosis. At present, SPECT whole-body imaging is mainly used in clinical practice to understand whether bone metastasis occurs and the extent of bone metastasis. With the popularization of PET-CT in clinical applications, reports on its diagnostic value for bone metastasis have been increasing year by year, but the conclusions are not completely consistent (14, 15), which may be related to different primary tumors and the different natures of their bone metastases. This study retrospectively compared the diagnostic value of SPECT whole-body bone imaging and 18F-FDG PET-CT whole-body imaging for bone metastasis of colorectal cancer (16). The results showed that the sensitivity, specificity and accuracy of 18F-FDG PET-CT whole-body imaging for bone metastasis of colorectal cancer were much higher than those of SPECT whole-body bone imaging.

The type of bone metastasis of colorectal cancer and the principles of 18F-FDG PET-CT imaging and SPECT determine the differences in the uptake amounts of 18F-FDG and SPECT (16). 18F-FDG, as an analogue of glucose, is transported into the cell under the action of glucose transportor proteins and phosphorylated into 6-phosphate-FDG under the action of hexokinase. However, the latter cannot undergo further metabolism and remains in the cell, thus reflecting the metabolic activity of living cells. The imaging principle of metastatic tumors is based on the increase of glucose metabolism in tumor tissues. Therefore, the amount of 18F-FDG uptake by the lesion is higher than that of the surrounding normal tissues. For osteolytic bone metastases, the main component is that tumor cells destroy bone tissues and form masses locally. Therefore, the uptake of 18F-FDG by the lesion is higher than that of the surrounding bone tissues. Osteogenic bone metastasis is mainly characterized by active osteoblasts and increased osteogenesis at the lesion site. Therefore, the increase in 18F-FDG metabolism is not obvious. Mixed bone metastasis will increase the uptake of 18F-FDG (17).

The principle of SPECT in diagnosing bone imaging is that 99MTC-labeled phosphate compounds combine with the surface of hydroxyapatite crystals and bone collagen fibers in organic matter through chemical adsorption and deposit in the bone. Mature bone collagen fibers can also directly absorb 99MTC-labeled phosphic acid compounds, causing the bone tissue to aggregate with radioactive imaging agents for imaging. When the metabolism and renewal of inorganic salts in bone lesions are vigorous, local blood flow increases, osteoblasts become active and new bone forms, more osteogenic radioactive imaging agents can accumulate compared to normal bone, presenting a radioactive “hot zone”. Conversely, when the blood supply to the affected bone tissue is reduced or interrupted, or when the bone salt metabolism is mainly the osteolytic process with almost no osteogenic effect, the aggregation of bone imaging agents in this area decreases accordingly, or even there is no aggregation of imaging agents, then a radioactive “cold zone” occurs. The sensitivity of SPECT whole-body bone imaging in detecting radioactive “cold zones” is significantly lower than that in detecting “hot zones” (18).

The types of bone metastasis of colorectal cancer are mostly osteolytic changes, a few are osteogenic changes, and some are mixed changes. Therefore, the sensitivity and accuracy of 18F-FDG PET-CT in detecting bone metastasis lesions of colorectal cancer increase, while the detection rate of osteolytic lesions of colorectal cancer by SPECT whole-body bone imaging decreases. This study found that the characteristics of the lesions detected jointly by CT, PET and SPECT were that the lesion range was large (generally > 1 cm), and the lesions mostly showed mixed changes on CT, while for simple osteolytic lesions with smaller lesions, SPECT was mostly negative. Therefore, for bone metastases of colorectal cancer mainly of the osteolytic type, 18F-FDG PET-CT examination is superior to SPECT imaging. However, for bone metastases mainly of the osteogenic type (such as prostate cancer), SPECT has obvious advantages. Clinically, different examination methods should be selected according to different primary tumors (19).

18F-FDG PET-CT has significant advantages in the diagnosis of bone metastases from colorectal cancer. However, the diagnosis of bone metastases by 18F-FDG PET-CT is significantly affected by radiotherapy and chemotherapy. After radiotherapy and chemotherapy, the activity of tumor cells is inhibited, the metabolic level decreases, and the ability to take up 18F-FDG decreases, which may lead to false negative results. Meanwhile, for osteogenic bone metastases, SPECT imaging should also be combined to avoid misdiagnosis. In conclusion, in the clinical practice process, it is necessary to closely combine the patient’s medical history as well as the location and type of the primary tumor to select the appropriate examination method. In addition, due to the high price of 18F-FDG PET-CT, it is not yet possible to conduct routine detection of tumor bone metastasis. Therefore, SPECT imaging remains a routine method for diagnosing bone metastasis. However, it should be noted that for patients with negative SPECT examination but highly suspected bone metastasis clinically, further examinations such as MR And PETCT should be performed to avoid missed diagnoses.

Accordingly, for routine initial staging of asymptomatic colorectal cancer patients without skeletal-related events, 18F-FDG PET-CT is not recommended as a universal first-line examination due to its relatively high cost and limited availability, particularly in resource-limited medical centers. PET-CT nevertheless provides important additional value for asymptomatic high-risk patients, such as those with advanced T/N stage, elevated tumor markers, or poorly differentiated histology, in whom early detection of occult bone metastases directly affects clinical staging, treatment selection, and prognostic evaluation. In comparison, SPECT whole-body bone imaging remains an appropriate and cost-effective first-line screening method for routine initial staging of asymptomatic colorectal cancer patients owing to its wide accessibility and low cost. For asymptomatic patients with negative SPECT results but high clinical suspicion of bone metastasis, or those who require accurate staging to guide treatment strategies, further examination with PET-CT or MRI should be performed to reduce false-negative outcomes and avoid tumor understaging.

However, several limitations should be acknowledged in this study. First, this was a retrospective single-center study with a relatively limited sample size. Second, the diagnostic analysis was mainly lesion-based rather than patient-based, and multiple lesions from the same patient may not be completely statistically independent. Third, pathological confirmation of bone lesions was unavailable because bone biopsy is rarely performed in routine clinical practice for colorectal cancer bone metastases.

Although 18F-FDG PET-CT demonstrated superior diagnostic performance in the present study, SPECT remains a widely available and cost-effective first-line screening tool for bone metastasis in routine clinical practice. In many medical centers, especially in resource-limited settings, PET-CT may not be routinely accessible because of its relatively high cost. Therefore, SPECT and PET-CT should be considered complementary imaging modalities rather than mutually exclusive diagnostic approaches.

## Data Availability

The raw data supporting the conclusions of this article will be made available by the authors, without undue reservation.
